# Effects of common anesthetic agents on [^18^F]flumazenil binding to the GABA_A_ receptor

**DOI:** 10.1186/s13550-016-0235-2

**Published:** 2016-11-08

**Authors:** Mikael Palner, Corinne Beinat, Sam Banister, Francesca Zanderigo, Jun Hyung Park, Bin Shen, Trine Hjoernevik, Jae Ho Jung, Byung Chul Lee, Sang Eun Kim, Lawrence Fung, Frederick T. Chin

**Affiliations:** 1 Departments of Radiology, Stanford University School of Medicine, Stanford, CA USA; 2 Psychiatry & Behavioral Sciences, Stanford University School of Medicine, Stanford, CA USA; 3 Neurobiology Research Unit, Rigshospitalet, Copenhagen, Denmark; 4 Department of Molecular Imaging and Neuropathology, New York State Psychiatric Institute, New York, NY USA; 5 Department of Psychiatry, Columbia University, New York, NY USA; 6 The Intervention Centre, Oslo University Hospital, Oslo, Norway; 7 The Norwegian Medical Cyclotron Centre, Oslo, Norway; 8 Bio Imaging Korea Co., Ltd, Seoul, Republic of Korea; 9 Department of Nuclear Medicine, Seoul National University College of Medicine, Seoul National University Bundang Hospital, Seongnam, Republic of Korea; 10 Center for Nanomolecular Imaging and Innovative Drug Development, Advanced Institutes of Convergence Technology, Suwon, Republic of Korea; 11 Department of Transdisciplinary Studies, Graduate School of Convergence Science and Technology, Seoul National University, Seoul, Republic of Korea; 12 Department of Radiology, Cyclotron Radiochemistry, Molecular Imaging Program at Stanford, Stanford University School of Medicine, 1201 Welch Road, Rm PS049, Stanford, CA 94305-5484 USA

**Keywords:** Flumazenil, GABA_A_ receptor, PET, Anesthesia, Ketamine, Isoflurane, Dexmedetomidine

## Abstract

**Background:**

The availability of GABA_A_ receptor binding sites in the brain can be assessed by positron emission tomography (PET) using the radioligand, [^18^F]flumazenil. However, the brain uptake and binding of this PET radioligand are influenced by anesthetic drugs, which are typically needed in preclinical imaging studies and clinical imaging studies involving patient populations that do not tolerate relatively longer scan times. The objective of this study was to examine the effects of anesthesia on the binding of [^18^F]flumazenil to GABA_A_ receptors in mice.

**Methods:**

Brain and whole blood radioactivity concentrations were measured ex vivo by scintillation counting or in vivo by PET in four groups of mice following administration of [^18^F]flumazenil: awake mice and mice anesthetized with isoflurane, dexmedetomidine, or ketamine/dexmedetomidine. Dynamic PET recordings were obtained for 60 min in mice anesthetized by either isoflurane or ketamine/dexmedetomidine. Static PET recordings were obtained at 25 or 55 min after [^18^F]flumazenil injection in awake or dexmedetomidine-treated mice acutely anesthetized with isoflurane. The apparent distribution volume (V_T_*) was calculated for the hippocampus and frontal cortex from either the full dynamic PET scans using an image-derived input function or from a series of ex vivo experiments using whole blood as the input function.

**Results:**

PET images showed persistence of high [^18^F]flumazenil uptake (up to 20 % ID/g) in the brains of mice scanned under isoflurane or ketamine/dexmedetomidine anesthesia, whereas uptake was almost indiscernible in late samples or static scans from awake or dexmedetomidine-treated animals. The steady-state V_T_* was twofold higher in hippocampus of isoflurane-treated mice and dexmedetomidine-treated mice than in awake mice.

**Conclusions:**

Anesthesia has pronounced effects on the binding and blood-brain distribution of [^18^F]flumazenil. Consequently, considerable caution must be exercised in the interpretation of preclinical and clinical PET studies of GABA_A_ receptors involving the use of anesthesia.

## Background

**Fig. 1:**
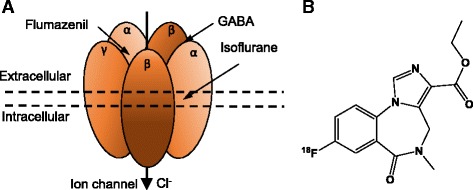
Structure of [^18^F]flumazenil (**a**) and the GABA_A_ receptor with different binding sites (**b**). The GABA binding site is located at the interface between α and β subunits while flumazenil and other benzodiazepines act as positive allosteric modulators on a distinct binding site between the α and γ subunits

Type-A γ-aminobutyric acid (GABA_A_) receptors are the principal inhibitory neurotransmitter receptors in the mammalian central nervous system (CNS) [1, 2], and are an important site of action for anesthetic agents and sedative drugs. However, there is insufficient information about the extent of occupancy at GABA_A_ sites affected by anesthetic agents. GABA_A_ receptors are pentameric ligand-gated chloride channels, belonging to the Cys-loop family of receptors [3] and comprised of several different subunit classes (α1–6, β1–4, γ1–3, δ, ε, π, ρ1–3) [4]. GABA, the endogenous agonist of GABA_A_ receptors, binds at an orthosteric site located at the interface between α and β subunits (Fig. 1a) [5, 6] while benzodiazepines (including isoflurane), a clinically important class of sedatives and anxiolytics, act as positive allosteric modulators by interacting with a distinct binding site between the α and γ subunits [4, 6]. A subset of GABA_A_ receptors containing α4 or α6 subunits are benzodiazepine insensitive [5]. Many radioligands have been developed for molecular imaging by positron emission tomography (PET), which target different sites of the GABA_A_ receptors ([6]). Of these, flumazenil (Ro 15-1788, Fig. 1b), a GABA_A_ receptor antagonist, has found the widest application in PET studies. Both [^11^C]flumazenil and the longer-lived isotopologue [^18^F]flumazenil have been used to measure the in vivo receptor availability in humans [7, 8], monkeys [9, 10], and rodents [11, 12].

GABA_A_ receptors are very important for cognitive function [13], and are implicated in the pathologies of conditions such as anxiety disorders [14, 15], schizophrenia [16, 17], and fragile X syndrome [18, 19]. Molecular imaging studies in humans are typically performed without anesthesia, but sedation may be required in patient populations who are unable to remain still for the duration of the scan. Preclinical imaging experiments in rodents are also commonly carried out under general anesthesia to minimize animal motion during the scan [20]. This introduces the possibility of pharmacological confounds arising from effects of the anesthetic agent on radioligand binding [21–27]. Selection of an appropriate anesthetic regimen is especially important for small animal PET imaging of GABA_A_ receptors, as pharmacologically and structurally distinct classes of general anesthetics (e.g. isoflurane, propofol, and pentobarbital) are all affecting the GABA_A_ receptor in either direct or indirect pathways [28]. Anesthesia can therefore potentially confound the results of [^11^C]flumazenil PET studies of GABA_A_ receptors in anesthetized humans or animals. Previous studies have reported that anesthesia with isoflurane [29] or propofol [30] increased the distribution volume (V_T_) of [^11^C]flumazenil in human brain. Furthermore, treatment with the membrane GABA transporter (GAT1) blocker tiagabine, which increases interstitial GABA levels, likewise increased the [^11^C]flumazenil V_T_ in the human brain [31], suggesting a positive allosteric potentiation of radioligand binding.

The NMDA-type glutamate receptor antagonist ketamine holds promise as a suitable anesthetic for GABA_A_ receptor PET studies, as subanesthetic ketamine doses were shown to have minimal effect on [^11^C]flumazenil binding in the human brain [32]. Furthermore, ketamine treatment did not increase interstitial GABA levels in an MR spectroscopy study in healthy humans [33], however, more recent studies showed increases in humans with obsessive compulsive disorder [34] and major depressive disorder [35]. The α_2_-adrenergic agonist dexmedetomidine is an anxiolytic agent often used to sedate children undergoing MRI scans [36]; however, synergistic interactions between α_2_-adrenergic agonists and benzodiazepine binding sites on GABA_A_ receptors have been described [37]. For example, a pilot study in healthy humans showed that dexmedetomidine sedation evoked a slight (4.9–6.0 %) increase in the grey matter binding potential (BP_ND_) of [^11^C]flumazenil [19].

The aim of this study was to explore the effects of the common anesthetic or sedative agents isoflurane, ketamine/dexmedetomidine, and dexmedetomidine alone on [^18^F]flumazenil binding to GABA_A_ receptors, in order to guide the design of pre-clinical and clinical imaging studies with this radiotracer. We used a combination of in vivo PET and ex vivo dissection to compare [^18^F]flumazenil binding in groups of mice with or without anesthesia in order to define optimal conditions for pre-clinical and clinical imaging.

## Methods

### Radiochemistry

All chemicals were from commercial vendors in pharmaceutical grade. Production of [^18^F]flumazenil was based on the method by Moon et al. [38]. [^18^F]fluoride in [^18^O]water was produced by a medical cyclotron (PETtrace-18, GE Healthcare) and passed through a PS-HCO_3_ cartridge, and eluted with a K222/K_2_CO_3_ phase transfer catalyst solution in the chemical module (Tracelab FX-_FN_, GE Healthcare). After azeotropic evaporation, 4 mg of flumazenil diaryliodonium tosylate salt precursor (Bio Imaging Korea Co., Ltd., Seoul, South Korea) in 1 mL of DMF with Tempo (1 mg) was added to the reactor. Fluorination proceeded for 5 min at 150 °C, whereupon the reaction mixture was diluted with 10 mL of water and passed twice through a C18 plus cartridge (Waters). The radiolabeled product was then eluted from the C18 cartridge with acetonitrile and diluted with sterile water. The crude solution was purified on a Luna C18 semi-preparative reversed-phase HPLC column (Phenomenex, 5 μm, 250 × 10 mm) with a mobile phase (acetonitrile/water = 20:80) delivered at 5 mL/min. Purified [^18^F]flumazenil was collected as the radiochemical peak eluting at 22 min, in a round bottom flask prefilled with 25 mL water for formulation. The diluted fraction was passed through the C18 plus cartridge and rinsed with water. The product was eluted with 1.5 mL of 100 % USP grade ethanol and diluted by 15 mL of USP grade saline, giving a final concentration below 10 % ethanol in sterile physiological saline. [^18^F]flumazenil showed ≥99 % radiochemical purity, specific activity of 264 ± 100 GBq/μmol (*n* = 3) at end of synthesis (EOS), and a decay-corrected radiochemical yield of 34 ± 22 % (*n* = 3).

### Animals

**Fig. 2:**
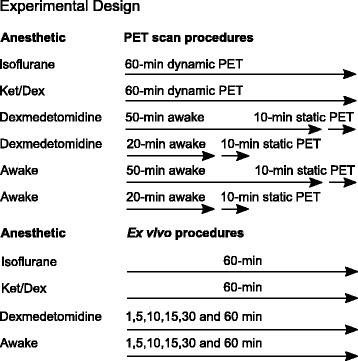
Experimental design of PET imaging (*n* = 4 in each scanning) and ex vivo (*n* = 4 at each time-point) procedures. A 10-min static PET scanning was obtained under acute isoflurane anesthesia, after an uptake phase while awake. Ket/Dex refers to a combined ketamine/dexmedetomidine dex anesthesia. All PET scanning groups were studied ex vivo at the end of the scan

Male mice (FBV/NJ, 5–6 weeks old, *n* = 45, weight at the time of scan 25 ± 2 g) were purchased from Jackson laboratories (Jax, Sacramento, CA). FBV/NJ male mice were chosen to support future studies in juvenile transgenic Fragile X KO FBV/NJ mice using [^18^F]flumazenil. All animal experiments were approved by the Stanford Administrative Panel on Laboratory Animal Care (APLAC) who is accredited by the Association for the Assessment and Accreditation of Laboratory Animal Care (AAALAC International) and uphold all federal and state regulations governing the humane care and use of laboratory animals. Mice had free access to food and water and were allowed ample time to acclimatize before the experiments. The mice were randomly assigned to one of four groups (isoflurane-treated, ketamine/dexmedetomidine (ket/dex)-treated, dexmedetomidine-treated (dex), and awake) and examined by a combination of PET and ex vivo procedures as outlined in Fig. 2. The isoflurane and ket/dex groups were subjected to a 60-min dynamic PET scan and then dissected for ex vivo biodistribution measurements. The dex and awake groups were scanned with PET for 10 min under brief isoflurane anesthesia at 20–30 (midframe at 25 min) or at 50–60 (midframe at 55 min) min post injection of [^18^F]flumazenil, followed by immediate ex vivo dissection. Furthermore, additional mice in the dex and awake groups were sacrificed for ex vivo biodistribution measurements at 1, 5, 10, 15, 30 or 60 min post injection of [^18^F]flumazenil. The drugs and respective dosages used for anesthesia were based on the recommended drug doses prescribed by IACUC—Institutional Animal Care and Use, by the AALAS—American Association of Laboratory Animals*.*

### PET acquisition

On the day of the experiment, the mice were transported to the imaging facility at least 1 h prior to the experiment. Animals were anesthetized one at a time using either 2 % isoflurane inhalation (*n* = 4), the ket/dex mixture (25 mg ket and 0.25 mg dex per mL sterile physiological saline; 4 mL/kg by intraperitoneal (i.p.) injection; *n* = 4), dex (0.25 mg/mL in sterile physiological saline; 80–120 μL by i.p. injection), and the last group was injected awake and without sustained anesthetic treatment. A catheter was placed in a tail vein of the fully sedated mice (isoflurane and ket/dex groups); in addition, an i.p. catheter was placed in the ket/dex anesthetized mice in order to give supplemental injection of 1 μL/g of the anesthetic mixture at 40 min after the initial dose. A bolus of 100 μL saline was injected in the tail catheter to maintain patency of the catheter and to hydrate the mouse. An ophthalmic lubricant was applied to the eyes of the animals. Mice undergoing PET scans were placed in a custom-made 4 × 4 mouse bed [39], which was positioned within the aperture of the Siemens Inveon microPET/CT. Isoflurane anesthetized and ket/dex anesthetized mice were scanned on separate days. The mice were kept warm during the scan using an infrared lamp. A CT scan was performed, for attenuation correction and anatomic registration, followed by a 60-min-long dynamic PET acquisition initiated upon intravenous bolus injection of [^18^F]flumazenil (5.30 ± 0.33 MBq, *n* = 45, within 2 h from EOS). The awake mice were placed in a tail-restraining device, and a glove containing warm water was applied to the restrained tail, which was then cleaned with 70% ethanol wipes. [^18^F]Flumazenil was injected as a bolus though a tail vein, and the animals were returned to their holding cage. At 25 or 55 min post tracer injection, the awake mice were briefly anesthetized with 2% isoflurane in oxygen at 2 L/min. The mice were positioned in the Siemens Inveon microPET/CT as described above, whereupon a 10-min PET acquisition was obtained, followed by a CT scan. At the conclusion of the scans, mice were sacrificed and radioactivity concentrations were measured in the brain, plasma, and whole blood as described below, corresponding to 35 and 65 min post injection, respectively.

### Ex vivo dissection

On the day of the experiment, the mice were transported to the imaging facility at least 1 h prior to the experiment. One group of mice was administered dex (1 mg/kg i.p.) as a single injection from a stock solution of 0.25 mg/mL (80–120 μL) in physiological saline, the other group received no pre-treatment. Each mouse was injected with [^18^F]flumazenil (5.30 ± 0.33 MBq, *n* = 45, within 2 h from EOS) while conscious and allowed to return to their home cage. The mice were sacrificed by cervical dislocation at times ranging from 1 to 60 min post [^18^F]flumazenil injection. Trunk blood samples were collected, and the brain was immediately removed from the skull and dissected into frontal cortex, striatum, hippocampus, brainstem, and cerebellum. A portion of trunk blood was centrifuged to separate the plasma. Radioactivity concentrations were measured in weighed tissue samples, whole blood, and plasma using a Packard Cobra II automated gamma counter, with correction to radioactivity at the time of tracer injection relative to the entire injected dose (%ID/g).

### PET/CT reconstruction

CT images were recorded for the whole field of view, using half rotation and 120 projections, 80 kV 500 μA current, and a 255 ms exposure. The CT image was reconstructed using a Feldman filtered back projection with 2× down-sampling and 4 × 4 binning, giving an effective pixel size of 103.2 μm. This structural image was used for attenuation correction of the PET recordings, as well as colocalization with the structural MRI. Decay events were recorded in list mode continuously over the course of the scan (60 or 10 min) with an energy window of 350–650 keV, and histogrammed into 1 × 10 min frames for static imaging or a series of 34 frames 12 × 10 s, 13 × 60 s, and 9 × 300 s) without scatter correction or smoothing. The frames were then reconstructed using four iterations of Fourier rebinning, followed by an attenuation-weighted two-dimensional ordered-subsets expectation-maximization (AW-2DOSEM) with a matrix size of 128 × 128 pixels.

### MRI acquisition

One representative mouse was anesthetized with 2 % isoflurane in oxygen at a flow rate of 2 L/min, and scanned with a dedicated small animal 7.0 Tesla MR scanner (Varian Magnex Scientific). The instrument was equipped with a gradient insert with a bore size of 9 cm (Resonance Research Inc, 770 mT/m, 2500 T/m/s), an amplifier (Copley 266), and a console (General Electric Medical Systems). Coronal two-dimensional images were acquired from the brain of the mouse, using a spin echo (SE) pulse sequence, echo time (TE) of 70 ms, repetition time (TR) of 3000 ms, and a slice thickness of 500 μm. The acquired data was reconstructed into a stack of 18 256 × 256 pixel images. The MRI images were used as an anatomical guide for definition of the regions of interest on the PET image.

### Image analyses

PET/CT and MR images were imported into the Inveon Research Workspace. PET data were co-registered manually to the MR images using the CT images as an anatomic guide. Brain regions of interest (ROI)—hippocampus and frontal cortex—were drawn on the MR image and on the left ventricle of the heart using the CT image and the first PET image frame. Time-activity curves (TACs) were then extracted from each ROI and calculated as %ID/g. The static PET images were summed from 20–30 and 50–60 min post tracer injection prior to registration and extraction of the mean ROI activities.

### Quantification of regional outcome measures via kinetic analysis

There is no real reference region of [^18^F]flumazenil in the mouse brain, and we therefore applied a more complicated kinetic analysis using either an image-derived input function or whole blood tracer concentration. Mean [^18^F]flumazenil TACs in the two brain ROIs (hippocampus and the frontal cortex) and the left ventricle from the isoflurane and ket/dex groups were calculated by averaging of the individual recordings. For the ex vivo groups, the average [^18^F]flumazenil TACs in the two brain ROIs and for whole blood were obtained by averaging individually measured curves within the group. A parametric model defined by a straight line ascending to the initial peak followed by a tri-exponential decay was fitted to the mean heart TACs and the average whole blood curve. The image-derived blood curves were not corrected for the metabolism of [^18^F]flumazenil because multiple blood samples could not be performed in same mice. The contribution of vascular radioactivity to the regional brain target TACs was corrected prior to analysis, assuming a 5 % blood volume. The corrected target TACs in each dataset were then fitted using one-tissue compartment (1TC) and two-tissue compartment (2TC) models [40], with the fitted average heart TAC serving as an image-derived input function for the isoflurane and ket/dex groups, and the fitted average whole blood curve for the ex vivo awake and dex groups. Kinetic rate constants (*K*_1_ and *k*_2_ for 1TC and *K*_1_ to *k*_4_ for 2TC) of the compartmental models were estimated by nonlinear regression using MATLAB R2012b (Mathworks, Natick, MA, USA). Although TACs for both whole blood and plasma were available for the ex vivo and dex groups, the whole blood TAC was used as the input function as it was considered to be more directly comparable to the image-derived input function from the dynamic PET scans. The heart ventricle image-derived input function is commonly used for calculation of distribution volume for tracers in small animals where sequential blood sampling is not possible and where binding in myocardium is negligible [41, 42]. We denote here the estimates of the [^18^F]flumazenil apparent total volume of distribution (V_T_) obtained relative to the whole blood input function (image-derived input function) as V_T_*, to distinguish it from the (true) V_T_ obtained from metabolite corrected plasma concentration, as defined by Innis et al. [43]. The measured activity concentration in whole blood and plasma is similar so choosing the whole blood should not give any bias on V_T_* calculations. As a measurement of precision, standard errors were computed for each estimated V_T_* value using a bootstrap algorithm [41] that incorporates errors associated with fitting the input function and the TAC for each target ROI.

### Statistics

All values are given as value ± standard error of the mean. Significant differences are calculated using an unpaired two-sided *t* test unless otherwise noted, *p* values less than 0.05 are considered statistically significant and significance levels in graphs are marked as follows: *p < 0.05, **p < 0.01, and ***p < 0.005.

## Results

**Fig. 3:**
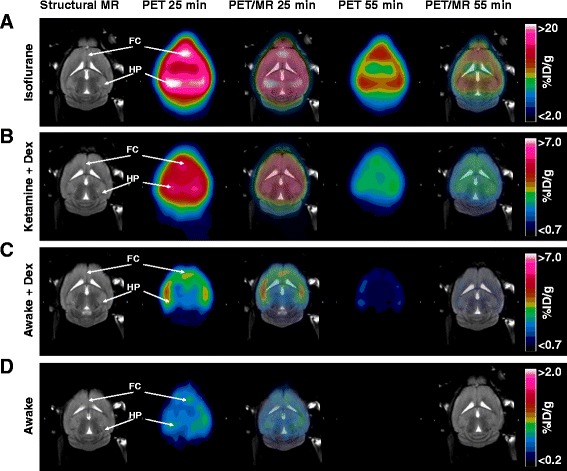
Representative PET images overlaid a reference MR T1 structural image for localization of regions. Summed images from a dynamic scan under isoflurane anesthesia (**a**) or ket/dex anesthesia (**b**) and static PET images from mice which have been injected awake then scanned under brief isoflurane anesthesia, treated with dex alone (**c**), and previous awake animals (**d**). Note that the *scale bars* are different for **a**–**d** in order to illustrate the full range of [^18^F]flumazenil binding

Static PET imaging under the four test conditions (Fig. 3) clearly depicts the effects of anesthesia on [^18^F]flumazenil binding to the GABA_A_ receptors. At 25 min post injection, the isoflurane anesthetized mice had uptake exceeding 20 %ID/g in the high binding regions of frontal cortex and hippocampus, compared with less than 2 %ID/g in the awake mice. The brain uptake in the ket/dex and dex are of intermediate magnitude, with up to 7 %ID/g in hippocampus and frontal cortex at 25 min. This phenomenon was even more pronounced at 60 min post injection, where the isoflurane anesthetized mice still had brain concentrations close to 10%ID/g, whereas no discernible [^18^F]flumazenil binding remained in brain of the awake mice.

**Fig. 4:**
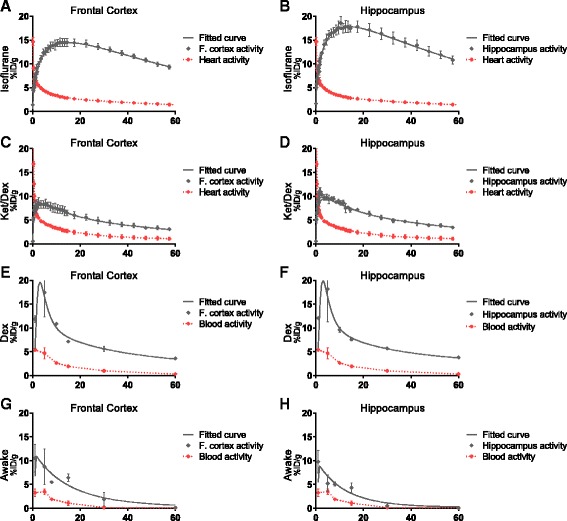
Time-activity curves from the frontal cortex and hippocampus during different anesthesia conditions, isoflurane anesthesia (**a, b**), ket/dex anesthesia (**c, d**) dex-treated awake mice (**e, f**), and awake (**g, h**). The *gray circles* represent the actual measured activity in the ROI, the *red circles* the image-derived input function or the ex vivo-derived input function from whole blood samples, and *corresponding lines* represents the fitted input curves

Brain TACs show that [^18^F]flumazenil uptake increases over the first 10–12 min, to a maximum of 19.1 ± 0.5 %ID/g in the hippocampus and 14.6 ± 0.9%ID/g in the frontal cortex in isoflurane-anesthetized mice (Fig. 4). The high brain levels of [^18^F]flumazenil persisted throughout the scan, with 11.4 ± 0.6 %ID/g in hippocampus and 9.3 ± 0.4 %ID/g in frontal cortex 60 min post injection. The left heart ventricle activity peaked at 6.2 ± 0.1 %ID/g very quickly after injection (~1 min post tracer injection) and declined rapidly thereafter. This rapid decline suggests that there is no specific binding in the myocardium. [^18^F]flumazenil uptake in ket/dex anesthetized mice peaked at 2 min post injection with 9.7 ± 1.3 %ID/g in hippocampus, and at 5 min with 8.4 ± 0.9 %ID/g in frontal cortex, whereas the heart signal peaked at 1 min with 7.9 ± 0.3 %ID/g and declined rapidly as in the isoflurane-anesthetized mice. Dex treatment alone sedated, but did not anesthetize the mice, such that dynamic PET acquisition was not possible in this treatment group. In the dex animals, [^18^F]flumazenil uptake peaked at 5 min post injection with 18.2 ± 6.9 %ID/g in the hippocampus, and at 17.5 ± 5.2%ID/g in frontal cortex, whereas the whole blood concentration peaked at 1 min with 5.4 ± 0.1 %ID/g. The [^18^F]flumazenil uptake in awake mice, as measured ex vivo*,* peaked at 1 min at 9.8 ± 2.4 %ID/g in the hippocampus and 10.5 ± 2.9 %ID/g in the frontal cortex, and the whole blood content also peaked at 1 min with 3.3 ± 0.8 %ID/g.

**Fig. 5:**
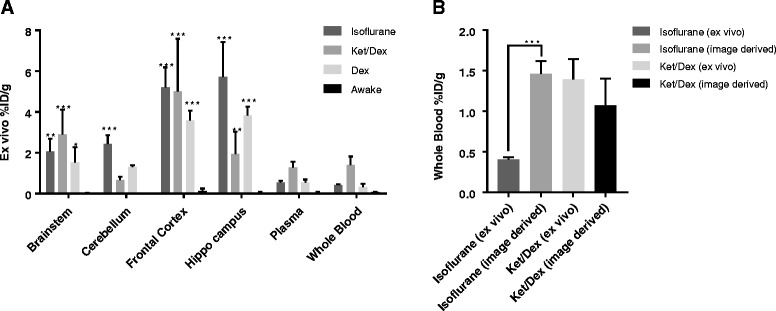
**a** Radioactivity concentrations in dissected brain regions and blood samples at 60 min post [^18^F]flumazenil injection, values are given as the %ID/g in the region of interest. **b** Comparison of the radioactivity concentrations in sampled whole blood (ex vivo) vs. the image-derived heart concentration from the PET scanner. Significant differences are reported relative to the awake condition

The [^18^F]flumazenil uptake measured ex vivo (Fig. 5) in hippocampus and frontal cortex was significantly greater at 60 min post injection in all anesthetized conditions compared with awake mice. The 2TC model consistently provided better fits of the target TACs than did the 1TC model. There was no statistically significant difference between the [^18^F]flumazenil V_T_* in the anesthetized and dex-treated conditions in these brain regions (frontal cortex *p* = 0.26, hippocampus *p* = 0.18). While uptake in the ket/dex anesthetized mice was very similar between the ex vivo and in vivo experiments, the uptake under isoflurane anesthesia was much lower when measured ex vivo relative to the in vivo PET uptake derived from the last frame 50–60 min post injection (frontal cortex, isoflurane ex vivo 5.2 ± 0.5 % ID/g vs. in vivo 9.3 ± 0.4 % ID/g, *p* < 0.001) (Fig. 5b)*.* Furthermore, of all the treatments, only isoflurane increased tracer uptake ex vivo in the cerebellum compared with awake mice. There was a similar trend towards an effect of isoflurane on radioactivity concentration in whole blood and plasma samples.

**Fig. 6:**
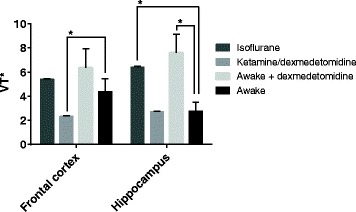
The apparent volume of distribution (V_T_*; mL/g^−1^) as calculated using the two-tissue compartment model with an input function but without metabolite correction. Significant differences are relative to the awake condition

The apparent distribution volume relative to the heart blood input (V_T_*) in the frontal cortex was unaffected by isoflurane or dex relative to the awake group, as shown in Fig. 6. However, combined ket/dex anesthesia did lower the V_T_* in the frontal cortex significantly (*p* = 0.008). However, V_T_* in the hippocampus was significantly increased by isoflurane (*p* < 0.001) and dex alone (*p* = 0.002) when compared to awake mice, whereas ketamine was without effect. Furthermore, there was a highly significant difference in V_T_* between the isoflurane condition and the ket/dex condition (*p* < 0.001) in both frontal cortex and hippocampus.

## Discussion

The PET image data presented in Fig. 3 clearly depict the effect of anesthetics on [^18^F]flumazenil uptake and binding to GABA_A_ sites in mouse brains: In the awake condition, there was no discernible radioactivity in the mouse brain at 60 min post injection, while all the anesthetic conditions showed a persistent signal throughout the brain. Isoflurane and other inhalation anesthetics have long been known to perturb [^18^F]flumazenil distribution in the brain. Gyulai et al. [29] showed that isoflurane globally increases uptake and binding of [^11^C]flumazenil in human brain in a dose-dependent manner, as much as doubling the distribution volume relative to the unanesthetized baseline. Also in humans, Salmi et al. [30] showed that sevoflurane, an anesthetic from the same chemical class as isoflurane, also increased the distribution volume of [^11^C]flumazenil by 15–20 % in all regions except the pons and white matter. In a test of a mixture of ketamine and isoflurane anesthesia in rhesus monkeys, Sandiego et al. [10] found a slight decrease in the [^11^C]flumazenil binding potential, possibly due to opposing effects of the two anesthetics. Our data with isoflurane alone support these previous findings; we find an up to 94 % increase in maximum uptake in hippocampus (19.1 % ID/g isoflurane versus 9.8 % ID/g awake), corresponding to a 128 % increase in V_T_* (6.4 mL/g isoflurane versus 2.8 mL/g awake). The direct comparison between the PET-derived data and ex vivo data is prone to bias induced by attenuation correction and partial volume effects as well as motion artifacts specifically in the ROI on the left ventricle; all these effects suggest that the V_T_* from PET might be overestimated compared with ex vivo measurements. The comparison PET to ex vivo is further complicated by administration of isoflurane in a chamber for the ex vivo studies versus a head cone in the in vivo procedure, resulting in slight dose differences. Nonetheless, we do find the same trend when comparing the ex vivo conditions at 60 min post injection; the [^11^C]flumazenil concentration ex vivo in the hippocampus was 5.7 % ID/g in the isoflurane condition versus only 0.048 % ID/g in the awake condition. Despite this remarkable confounding effect of anesthesia on [^11^C]flumazenil and [^18^F]flumazenil binding, some preclinical PET imaging studies of GABA_A_ receptors are still published using isoflurane anesthesia.

The combination ket/dex has been the anesthesia of choice in preclinical molecular imaging studies with flumazenil based on the report by Salmi et al. [32], who did not find any effect of subanesthetic doses of ketamine on [^11^C]flumazenil binding in humans. However, to our knowledge, this is the first such study to compare an anesthetic dose of the commonly used mixture ket/dex to the awake condition. We find that an anesthetic dose of the ket/dex mixture does not alter the initial peak uptake of [^18^F]flumazenil in the hippocampus (9.7 ± 1.3 % ID/g in ket/dex and 9.8 ± 2.4% ID/g in awake). However, it did significantly enhance the late retention of [^18^F]flumazenil in hippocampus, which was 1.95 % ID/g at 60 min post injection, versus only 0.048% ID/g in the awake condition. Dex alone did increase the initial uptake of [^18^F]flumazenil in the hippocampus by up to 85 % (18.2 % ID/g dex and 9.8 % ID/g awake), and had similar effects as the ket/dex mixture on tracer retention as measured ex vivo 60 min post injection. Interestingly, there was a 47 % decrease in the calculated frontal cortex V_T_* for the ket/dex (2.33 ± 0.05 mL/g) condition compared with awake animals (4.39 ± 1.06 mL/g) but no difference in the hippocampus, while dex alone increased V_T_* by 171% in the hippocampus (7.60 ± 1.55 mL/g), compared with awake animals (2.77 ± 0.72 mL/g) but with no such effect in the frontal cortex. The frontal cortical decrease in V_T_* with ket/dex anesthesia supports the findings of Sandiego et al. [10], who found a decrease in [^11^C]flumazenil binding potential (BP_ND_ with the pons as reference region) in ketamine/isoflurane-anesthetized Rhesus Monkeys. The higher hippocampal uptake in mice with dex treatment supports the findings of D’hulst et al. [19], who found a mild (4.–6.0 %) increase in the binding of [^18^F]- or [^11^C]flumazenil binding in dex-treated humans. This increase specifically in the hippocampus V_T_* by dex alone could explain why ket/dex did not decrease the V_T_* in both regions, as the effects could have been counteracted by the dex induced increase.

We observed a different response to the anesthetics comparing the uptake and distribution volume in the frontal cortex with the hippocampus. This could be explained by the different site of action between [^18^F]flumazenil and anesthetic and by the heterogenous distribution of GABA_A_ receptors subunits [44]. More than 20 different subunit combinations have been reported [4, 45], among these, α1 subunits are widely distributed in the cortex while α5 subunits are more pronounced in the hippocampus [44]. In particularl, Momosaki et al. compared the binding of [^3^H]flumazenil (RO15-1788) to that of the structurally related [^3^H]Ro15-4513, which is more α5 subunit selective than [^3^H]flumazenil [46], and found a remarkable difference in the GABA_A_ subtypes between the cortex and hippocampus. Furthermore, the GABAergic functionality is also different between cortical and hippocampal regions, Whissell et al. found that while the majority of cortical GABAergic neurons was parvalbumin (PV-expressing) positive, the hippocampal was cholecystokinin (CCK-expressing). While we cannot conclude on the exact reason for the regional difference base on our study, we do find other evidence of regional differences in GABA_A_ receptor positive neurons.

There are several limitations to this study. Biases from attenuation correction and partial volume effects can hinder the direct comparison of PET data and ex vivo data. We did see a difference in the whole blood radioactivity of the isoflurane-anesthetized group when we compare the ex vivo uptake values at 60 min with the PET imaging-derived values; this difference might lead to an underestimation of the true (V_T_*) and thus lower the significance in our results. Our index of distribution volume (V_T_*) is calculated using the whole blood radioactivity instead of the plasma radioactivity as an input function and is not corrected for plasma metabolites but assumes that whole blood activity remains as untransformed parent compound. The reported major metabolites of [^18^F]- or [^11^C]flumazenil are known polar compounds [47] and, as such, should not enter the brain. However, differences in metabolite rates could affect the radioactive concentration in blood and therefore is a potential bias on the V_T_* calculations. The heart ventricle image-derived input function serves for calculation of distribution volume for tracers without substantial binding in the myocardium and without important metabolism during the scan [48, 49]. Finally, we calculated V_T_* on a group basis rather than for individual mice, thus affording a better comparison of PET scans and the ex vivo experimental results.

We cannot rule out that some effects of anesthesia on [^18^F]flumazenil uptake and binding arose from changes in cardiac output, since central and peripheral GABA_A_ receptors do regulate heart rate [48], although cerebral blood flow is the more salient issue, since it can influence delivery of lipophilic tracers to the brain. We argue that the V_T_* index is a less biased measurement than %ID/g in this regard, as it is a steady-state index. Furthermore, [^18^F]flumazenil is a substrate of the blood-brain barrier efflux transporter P-glycoprotein [49], we cannot rule out that anesthetics might have an actions on P-glycoprotein function, further studies in P-glycoprotein knockout animals would be needed.

## Conclusions

We found that anesthesia or sedation with isoflurane, a ketamine/dexmetomidine mixture, and dexmetomidine alone all changed the pharmacokinetics of [^18^F]flumazenil, consistently increasing tracer uptake, relative to nonanesthetized condition (awake). Only the ketamine/dexmetomidine mixture did not change the volume of distribution (V_T_*) of [^18^F]flumazenil in the hippocampus, arguably due to opposing effects of the two agents in combination. Results from this study emphasize the importance of careful study design and control of the experimental conditions when imaging GABA_A_ receptors under a condition of anesthesia. Any group differences in [^18^F]flumazenil PET results need to be interpreted with caution due to the potentially confounding effects of anesthetic agents.

## Abbreviations

CT: Computerized Tomography
Dex: Dexmedetomidine
GABA_A_ receptor: Type-A γ-aminobutyric acid receptor
ID/g: Injected dose per gram
Ket/dex: Ketamine/dexmedetomidine
MRI: Magnetic resonance imaging
PET: Positron emission tomography
ROI: Region of interest
TAC: Time-activity curve
V_T_*: Distribution volume*
V_T_: Distribution volume
